# Type I Diabetes Mellitus: Genetic Factors and Presumptive Enteroviral Etiology or Protection

**DOI:** 10.1155/2014/738512

**Published:** 2014-12-10

**Authors:** Jana Precechtelova, Maria Borsanyiova, Sona Sarmirova, Shubhada Bopegamage

**Affiliations:** Enterovirus Laboratory, Faculty of Medicine, Slovak Medical University, Limbova 12, 83303 Bratislava, Slovakia

## Abstract

We review type 1 diabetes and host genetic components, as well as epigenetics and viruses associated with type 1 diabetes, with added emphasis on the enteroviruses, which are often associated with triggering the disease. Genus *Enterovirus* is classified into twelve species of which seven (*Enterovirus A, Enterovirus B, Enterovirus C,* and *Enterovirus D* and *Rhinovirus A, Rhinovirus B,* and *Rhinovirus C*) are human pathogens. These viruses are transmitted mainly by the fecal-oral route; they may also spread via the nasopharyngeal route. Enterovirus infections are highly prevalent, but these infections are usually subclinical or cause a mild flu-like illness. However, infections caused by enteroviruses can sometimes be serious, with manifestations of meningoencephalitis, paralysis, myocarditis, and in neonates a fulminant sepsis-like syndrome. These viruses are often implicated in chronic (inflammatory) diseases as chronic myocarditis, chronic pancreatitis, and type 1 diabetes. In this review we discuss the currently suggested mechanisms involved in the viral induction of type 1 diabetes. We recapitulate current basic knowledge and definitions.

## 1. History of Diabetes

Symptoms of type 1 diabetes (T1D) have been recognized since approximately 1500 BC, when they were described on Egyptian papyrus as indicators of a rare disease that caused patients to lose weight rapidly and experience “too great emptying of the urine” [1, 2]. This was probably the first mention of the disease. At approximately the same time, however, Indian physicians realized that the urine of some patients attracted ants. These doctors classified the disease and named it “madhumeha” or “honey urine” [3]. Later, the disease was called “diabetes” by Greek physician Aretaeus, who noted symptoms such as constant thirst, excessive urination, and loss of weight. “Diabetes” comes from the Greek word for “siphon” (to draw off or convey liquid). The Arabian physician Avicenna (980–1037) was the first to bring attention to the complexity and progression of the disease, recognizing primary and secondary diabetes. In the 17th century, the Latin term “mellitus” meaning “honeyed” or “sweet” was added by Thomas Willis, an English physician, in his treatise* Pharmaceutice Rationalis* (1674). He tested urine samples of patients to determine the presence of diabetes; those samples with a sweet taste indicated diabetes mellitus or “honeyed” diabetes. In 1776, Matthew Dobson measured the quantity of glucose in the urine samples of diabetic patients. Dr. Frederick Allen, a diabetes specialist in the early 20th century, advised low calorie diets for his diabetic patients. These diets increased the life of his patients, but they often became weak and starved [4].

A critical experiment occurred in 1921 when Frederick Banting and his assistant, Charles Best, kept a dog with diabetes alive for 70 days by injecting it with a turbid mixture of an extract from a canine pancreas. Dr. Collip and Dr. Macleod then helped Banting and Best to administer a more refined extract of insulin to Leonard Thompson, a boy suffering from diabetes. They noted that, within 24 hours, his high blood sugar had dropped to nearly normal levels. Banting and Best received the Nobel Prize in Physiology or Medicine in 1923 for their discovery of insulin. Since that time, scientific interest in this disease and associated glucose metabolism has increased. Ten scientists have received Nobel Prizes for diabetes-related investigations [5]. Sir Harold Himsworth reported the existence of two types of diabetes in 1935: “insulin sensitive” (type I) and “insulin insensitive” (type II). His work [6] was an important landmark in the understanding of diabetes and treatment strategies.

## 2. Types of Diabetes Mellitus

Diabetes mellitus (DM) is classified as a group of metabolic diseases with a typical clinical state of hyperglycemia (high blood glucose levels), which is an outcome of defective insulin secretion, insulin action, or both [7]. Hyperglycemia is correlated with typical acute diabetic symptoms that include excessive urine production (polyuria) and high urine sugar levels (glycosuria), the consequences of which are thirst, increased fluid intake (polydipsia), increased eating (polyphagia), weakness, fatigue, blurred vision, unexplained weight loss, and lethargy. Chronic hyperglycemia is further related to prolonged damage, dysfunction, and failure of different organs. Secondary complications due to fluctuations in blood glucose arise from vascular degeneration, which results in damage of eyes, kidneys, nerves, blood vessels, and heart, causing dysfunction and failure of the organs or eventual gangrene with a probable loss of toes, feet, and legs. These complications decrease the quality of life and can eventually lead to premature death.  Table 1 shows the recent classification of diabetes according to the American Diabetes Association and World Health Organization which includes four clinical categories of diabetes [7–9]. In this review we focus on type 1 diabetes because it is the most common endocrine disorder in children, with a worldwide increase in incidence.

Type 1 diabetes usually develops before the age of 30, with peaks at 2, 4–6, and 10–14 years of age. Type 1 diabetes patients depend on external insulin (usually injected subcutaneously) for survival. Approximately 5–10% of diabetics with type 1 diabetes (T1D) show gradual destruction of the insulin producing *β*-cells in the pancreas. This destruction leads to absolute insulin deficiency. The disease usually appears when 80–90% of the *β*-cells are destroyed [10, 11].

Autoimmune-mediated destruction of *β*-cells has been associated with T1D because 85–90% of the cases show one or more autoantibodies [12]. Some of the autoantigens described by different authors include enzyme glutamic acid decarboxylase (GAD65) [13], insulin (IAA) [14], insulinoma associated antigens (identified as tyrosine phosphatases IA-2 and IA2*β*) [15, 16], islet cell antigens (ICA-69) [17], enzyme carboxypeptidase-H [18], GM-gangliosides [19], 38 kD autoantigen [20], sex determining region-Y box protein (SOX13) [21], and zinc transporter 8 protein (ZnT8A) also known as the solute carrier family 30 member 8 (SLC30A8) [22]. The presence of two or more antibodies has been suggested as predictive markers for T1D [23, 24]. In the present times commercial kits are available and some laboratories have developed in-house methods for detection of some of these antibodies. The predictive value of these markers would be of importance for intervention measures. However, it is difficult to identify the right time to measure these antibodies. Another unclear aspect is whether these antibodies are produced after the cell destruction or if antibodies induce the cellular destruction.

Treatment of T1D usually consists of insulin administration via injections or pumps. Insulin therapy results in improvements, yet T1D is considered to be a chronic disease for which there is no prevention or cure. The disease progresses in the majority of diabetic patients, and the resulting dysglycemia leads to microangiopathic or neuropathic complications.

## 3. Type 1 Diabetes: Major Histocompatibility Complex Related Genetic Factors

Type 1 diabetes is a multifactorial disorder requiring a genetic predisposition and a trigger for the destructive process as observed in other autoimmune diseases [25, 26]. T1D has a strong genetic component. Relatives of diabetic patients have a high risk of developing the disease; siblings have a greater risk than offspring, and there is a high concordance rate among identical twins [27, 28]. This genetic predisposition (or lack thereof) is determined by the balance between susceptibility and resistance alleles. Among various factors the major histocompatibility complex (MHC) glycoproteins are very important in the recognition of the tissues by the immune system.

Genetic wide association studies (GWAS) help to identify and measure the DNA variations in the human genome and identify disease risk factors in a given population. This is done by measuring single nucleotide polymorphism (SNP) changes occurring frequently as single base pair changes in the DNA sequences [29, 30]. These variations are identified by genotyping using the next-generation sequencing and chip based microarray [31]. Epigenome wide association studies (EWAS), using this modern technology, have shown that, besides the HLA-A, HLA-B, and HLA-DP*β*1, other strong markers include HLA-DR*β*1, HLA-DQ*α*1, and HLA-DQ*β*1. The HLA-DR*β*1^*^03:01 haplotypes carrying HLA-DR*β*3^*^02:02 alleles showed a higher risk than HLA-DR*β*1^*^03:01 haplotypes carrying DR*β*3^*^01:01 in DR*β*1^*^03:01/^*^03:01 homozygotes with two DR*β*3^*^01:01 alleles [31–35].

The human leukocyte antigen (HLA) region of the 6p21 chromosome encodes glycoprotein of the MHC, which has a function in presenting antigenic peptides to T-cells. MHC is a cluster of genes that are situated on the short arm of chromosome 6 and vary in length, depending on their haplotypes. The MHC locus first discovered by Snell and Higgins [36] comprises 121 functional genes, which include all of the MHC class I and MHC class II genes. MHC I molecules constitutively expressed in most cells present antigens for binding to CD8+ T-cells and display peptides from proteins synthesized within the cell. On the other hand MHC II molecules constitutively expressed in antigen-presenting cells (APCs), such as macrophages, dendritic cells, and B-cells, are important to the human immune response because they present peptide antigens to T-helper (CD4+) cells, revealing peptides from engulfed proteins present in thymic stromal cells or antigen-presenting cells [37–39]. MHC II molecules are also inducible in some tissues by cytokines such as interferon gamma (IFN-*γ*) [40].

**Figure 1:**
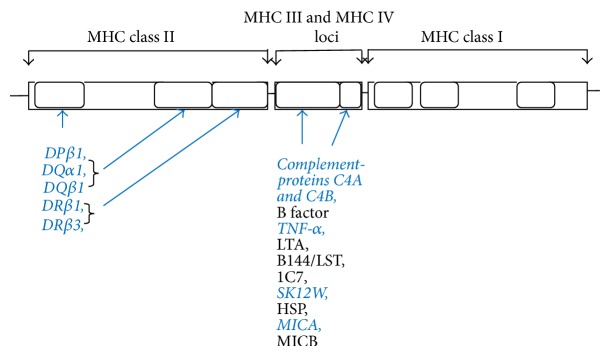
Major histocompatibility complex loci associated with type 1 diabetes. The known loci and products participating in the triggering of type 1 diabetes are marked in blue and italics.

In humans MHC I molecules comprise the HLA-A, HLA-B, and HLA-C while MHC II molecules include HLA-DP, DQ, and DR, which have the strongest association with T1D as summarized in Figure 1 and Table 2. Two types of MHC II genes encode alpha polypeptides and beta polypeptides and together form the functional class II alpha-beta heterodimer. They form major DP*α*, DP*β*, DQ*α*, DQ*β*, DR*α*, and DR*β*, plus minor DM and DO genes that encode MHC II proteins on the APCs. Both the alpha and beta polypeptide genes are polymorphic [37–43]. These proteins are important because of their role in triggering of autoimmune and inflammatory responses.

Autoimmune diseases such as systemic lupus erythematosus and psoriasis have been linked to MHC III and MHC IV loci [43, 44] but so far association of these loci with the T1D has not been investigated and remains a novel area for research. MHC III region is highly conserved but is more heterogeneous than MHC I and MHC II regions. The MHC III genes are located between the MHC I and MHC II on the short arm of chromosome 6 [43]. Figure 1 and Table 2 summarize the location of these loci. The human MHC III region contains 61 genes which encode the MHC class III molecules. These are proteins which are components of the immune and complement system such as C2, C4, and B factor. Lastly, MHC IV genes are involved in the production of several factors: tumor necrosis factor alpha (TNF-*α*), lymphotoxin alpha (LTA) and lymphotoxin beta (LTB), B144/LST protein expressed in dendritic cells and involved in their morphogenesis, 1C7 expressed in the natural killer (NK) cells and responsible for their activation, the G1 and allograft inflammatory factor 1 (AIF1) which are inflammatory markers, superkiller 12W (SK12W) that confers antiviral activity, B associated transcript (BAT1) encoding a protein with helicase motifs, heat shock proteins (HSP) related to proinflammatory cytokines and protection against cellular inflammation/apoptosis, and MIC A and MIC B genes coding for the MIC A and MIC B proteins which play a role in activation of natural killer cells [44]. The MHC III and MHC IV genes are involved in the antigen processing (but not presenting) and production of proinflammatory cytokines.

The influence of a particular HLA molecule on susceptibility to any disease depends on its three-dimensional structure [45, 51, 52]. The diabetogenic and protective molecules differ in structure. The differences result in varied antigen peptide selectivity, binding affinity, and the stability of the HLA molecule presented on the cell surface. The HLA molecules react with distinct peptide binding motifs and interact differently with a given diabetogenic autoantigen. The disease susceptibility conferred by HLA represents the combined effect of several genes within the MHC. At least three major loci are involved (HLA-DR*β*1, HLA-DQ*α*1, and HLA-DQ*β*1), but several other genes may also contribute [46, 53].

Susceptibility to T1D is most strongly determined by DQ*β*1 and I-A*β* equivalent chain allele for MHC in mice that encode serine, alanine, or valine at position 57 on both chromosomes. In contrast DQ*β* and I-A*β* in mice at position 57 aspartic acid positive alleles mediate resistance to T1D. To some extent, resistance is also mediated by DR*β*1 and I-E*β* in mice expressing aspartic acid at position 57 or glutamine at position 74 [47]. It has been suggested that HLA-DQ and HLA-DR polymorphism affects the susceptibility to T1D through the selectively affecting nature of the peptides presented to T-cells [48]. One copy (allele) of the DR3 or DR4 is found commonly in the general population. Individuals, susceptible to T1D, inherit two alleles DR3/DR3, DR4/DR4 or the high risk DR3/DR4 combination [49, 50] (Table 2). Heterogeneity in these alleles may increase or decrease the disease risk.

## 4. Epigenetics

Epigenetics is a study of heritable changes which are not a consequence of mutations in the DNA but occur as alterations at the transcriptional level. Increasing knowledge of the immune mechanisms that have the largest impact on the disease, modern molecular technology, and recent developments in the understanding of meta-analysis and epigenetics show that the T1D risk is a combination of reactions or a dialogue among four major factors: (1) primary T1D-related genetic susceptibility genes; (2) triggering immunological factors that may vary and act as stimuli; (3) other factors such as epigenetic heterogeneity caused by environmental factors; and (4) interactions with genes related to other (immunological and apoptotic) pathways expressed locally in pancreatic *β*-cells. Each of these factors has been reviewed elsewhere in recent years [35].

In addition to inherited alleles, other mechanisms regulating gene expression include “parent-of-origin effects” (marker from either the maternal or paternal allele), which can modify the inheritance and/or transcription of susceptibility genes [54]. The “parent-of-origin effects” show the differential behavior of the genes depending on the parent from whom they were inherited and relating them to different diseases [55]. The influence of the susceptibility to T1D in the offspring is stronger and more frequent to the father than the mother ranging from 6 to 9% [56–58]. The “parent-of-origin” susceptibility transmission also varies for different antibodies, with greater prevalence in children from diabetic fathers than mothers [59]. Alteration of DLK1-MEG3 gene region on chromosome 14q32.2 in the father appears to influence the susceptibility to T1D [60].

GWAS identified epigenetic modifications, which are changes in gene expression, and gene function without changes in the gene sequence. The three mechanisms defined include (1) the methylation of cytosine residues in DNA at position 5 (moving clockwise from NH_2_, which is counted as position 0) of the 6-atom ring of cytosine, associated with transcriptional repression or posttranslational modification; (2) N-terminal histones that may be affected by posttranslational modifications through acetylation, methylation, or phosphorylation; and (3) demethylation of miRNA. Rakyan et al. [61] analyzed DNA methylation variable positions (MVPs) for T1D on monocytes of monozygotic twins discordant for T1D. The MVPs were sought in these monozygotic twins in order to rule out typical genetic factors. The authors [61] showed that the methylations were not an effect of the disease but occurred prior to the disease.

## 5. Non-MHC Complex Genes Related to T1D

Since the first association of MHC and T1D [26, 62], a number of other non-MHC-related genes and immune mediators have been associated with T1D. Such genes include the insulin gene, cytotoxic T-lymphocyte antigen 4 gene (CTLA4) [26, 63, 64], protein tyrosine phosphatase N22 gene (PTPN22) [65], IL-2 receptor alpha (IL-2RA) [66], and interferon induced with helicase C domain 1 (IFIH1) gene [29]. GWAS studies and the non-MHC genes related to T1D have been discussed and compared by Bakay et al. [67].

Nejentsev et al. [68] identified four variations in the interferon induced with helicase C domain 1 (IFIH1) gene, which might be associated with reduced risk of developing T1D. The helicase enzyme IFIH1 (also known as MDA5 or melanoma differentiation-associated protein-5) triggers the secretion of interferons in response to viral infection. The interferon-regulating factor 7- (IRF7-) driven inflammatory network (IDIN) genes also contribute to the risk of T1D [69].

Different non-MHC candidate gene products related to T1D involving the cytokine pathways are IL-12B, 2′-5′-oligoadenylate synthetase 1 (OAS1), small ubiquitin-like modifier 4 (SUM04), paired box gene 4 (PAX4), protein tyrosine phosphatase N2 gene (PTPN2), regulatory and proinflammatory T-cells, macrophage-related cytokines including interferon gamma (IFN-*γ*), tumor necrosis factor alpha (TNF-*α*), interleukin-4 and interleukin-10 (IL-4 and IL-10), and C-type lectin domain family 16 (CLEC16A) [29, 69–81].  Table 3 shows the overview of the non-MHC genes and gene products associated with T1D and the chromosomes on which they are located. Although the functions of each of these products are well known in the regulation of the immune system (Table 3) their exact role inducing T1D remains unclear.

The multigenic nature of T1D has been recognized through meta-analysis studies [73, 82] and genetic epidemiology [83, 84]. To date several MHC and immune-related genes associated with antigen presentation and inflammatory regulation appear to serve as risk factors for T1D.

## 6. Genetic Mechanisms for T1D Predisposition or Protection

The interaction between cellular, immune, and genetic factors determines if a particular (experimental) system is prone to protection or predisposition to T1D. Studies on the involvement of B-cells in the induction of diabetes are limited. B-cells are common in inflamed insulin producing pancreatic islets [85–87]. Single nucleotide polymorphism analysis indicates the following variants are associated with T1D and B-cell receptor (BCR) and B-cell differentiation: PTPN22, PTPN2, Src homology 2B3 adapter gene (SH2B3), and immunoregulatory cytokines IL-10, IL-19, and IL-20 [88, 89]. Peripheral B-cell proliferation was shown to be related to the cytokine IL-10, and the “IL2-IL21 T1D” locus was associated with IL-10 production by the memory B-cells and the autoreactive T-cells [89]. B-cell subsets that might be linked to autoimmune diseases and related views have been discussed by Yang et al. [90]. The regulatory B-cells (B-reg) influence the responses of regulatory T-cells, effector cells, and invariant natural killer T-cells (iNKT) and activate the dendritic cells through cytokines. These cytokines are mainly the IL-10 and tumor growth factors (TGF), together influencing T1D. Research related to B-cell subtypes and T1D is increasingly accumulating.

Normally islet *β*-cells express low levels of IFNs, but viral infections may result in high levels of IFN production, which further increase MHC I expression leading to high susceptibility to cytotoxic CD8+ T-cell recognition and destruction [91]. Therefore, normal IFIH1 gene-mediated immune responses activated by some viruses may stimulate autoimmunity against pancreatic *β*-cells. However, mutations in IFIH1 gene disrupt this mechanism inducing protection against T1D by production of protein product MDA5 with an impaired function [68, 92].

Interferons can be identified as type I (IFN-*α* and IFN-*β*), type II (IFN-*γ*), and type III (IFN-*λ*). Among type 1 interferons the best studied ones are interferons alpha (IFN-*α*) and beta (IFN-*β*). These interferons are induced via stimulation of different transmembrane and cytosolic receptors. The main receptors responsible for type I interferon induction in response to infections and double stranded RNAs (dsRNAs) are the RNA helicases retinoic acid inducible gene 1 (RIG-1) and melanoma differentiation-associated gene 5 (MDA-5). Type 1 interferons are produced by most cell types, including plasmacytoid dendritic cells [93]. IFN-*α* usually limits viral replication, but, on the other hand, together with inflammatory cytokines IL-1*β* and TNF-*α*, these interleukins can cause direct cellular damage [94]. *β*-cells of the pancreatic islets express IFN-*α* [95]. Sera of T1D patients have increased titers of IFN-*α* [96]. Of epidemiological relevance IFN-*α* in the plasma of patients with detectable enteroviral-RNA [97] has been found to be in high concentrations.

Type II interferons (IFN-*γ*) are produced by different cells types. They enhance the MHC class I and class II expression via the protein tyrosine kinase of the Janus family (Jak 1 and Jak 2) leading to phosphorylation of the tyrosine in STAT1 [98, 99]. Interferon gamma (IFN-*γ*) interacts with IFN-*α*. In autoimmune diseases, type I interferons induced by the plasmacytoid dendritic cells may upregulate or downregulate the IFN-*γ* which in turn affects the MHC expression and apoptosis [100]. Whereas type III interferons, also known as interferon lambda (IFN-*λ*), are restricted only to few cell types, they may affect the viral replication and reduce viral induced damage to tissues. This was observed in primary human pancreatic islet cells recently [101] leading to their protection from coxsackievirus infection. The IFN-*λ* and its association with T1D could be a new area for investigation.

The relevance of genetic factors modulating the expression and function of various types of interferons and other immune-related cytokines is related to their critical importance in controlling infections. Among the various possible infections agents, viruses have been the most studied and consistently associated with the mechanisms and predisposition to T1D. A growing body of literature on* in vitro* and* in vivo* experiments indicates that viruses may drive unspecific inflammatory or immune responses against pancreatic cells critical in blood glucose homeostasis.

## 7. Viruses and Predisposition to Type 1 Diabetes

Viruses, with their potential to induce innate and adaptive immune responses and local inflammation in target organs, are suspected of initiating autoimmune processes. The etiologic link between T1D and viruses is based on epidemiological, serological, and histological findings, as well as experimental* in vivo* and* in vitro* studies. These include the DNA viruses from families* Herpesviridae* and* Parvoviridae* and RNA viruses of families* Togaviridae*,* Paramyxoviridae*,* Retroviridae*, and* Picornaviridae* (Tables 4 and 5). The viral genetics, host genetics, host immunological status, age at the time of exposure to the virus, and the pancreatic microenvironment may influence triggering of T1D related to *β*-cell damage. A systematic review and meta-analysis of observational molecular studies in 2011 showed a strong association between T1D and enteroviruses [82]. Tables 4 and 5 show the different viruses and the mechanisms of induction of T1D. Also the proposed mechanisms related to the virus-induced impact on the pancreatic beta cells have been studied using animal/*in vitro* models and enteroviruses.

### 7.1. Human Cytomegalovirus

In 1979, the human cytomegalovirus (HCMV), also known as human herpes virus-5 (HHV-5), was first linked to T1D [124] onset following a congenital infection by this virus. These findings were confirmed by detection of cytomegalovirus genome in 22% of diabetic patients correlating with the presence of islet cell antibodies (ICA) [102]. The same group showed cross-reactivity of anti-cytomegalovirus antibodies with 38 kD human pancreatic islet-specific protein [125]. The virus infected human fetal islets* in vitro,* yet direct destruction of *β*-cells was absent [126]. The different mechanisms suggested for the role of human cytomegalovirus in diabetogenesis maybe related to (1) molecular mimicry induced by T-cell cross-reactivity between human cytomegalovirus and GAD65 (glutamic acid decarboxylase exists as two isoforms GAD65 and GAD67, a major enzyme required for the production of the gamma amino butyric acid which regulates the glucagon secretion) in pancreatic islet *β*-cells [102, 103]; (2) the persistence of HCMV specific CD4+ T-cells or a bystander activity [104]; and (3) persistent infection in *β*-cells [127]. However, other groups [128, 129] failed to find a link between the virus and T1D; no human cytomegalovirus DNA was found in the formalin-fixed pancreases of patients with T1D [130, 131]. The contribution of human cytomegalovirus to the diabetogenic process is not clear yet and controversial; more clinical and model studies related to this virus and T1D are required.

### 7.2. Parvovirus

Parvovirus B19 belongs to family* Parvoviridae*, genus* Erythroparvovirus*. The virus may affect individuals of any age, but the infections are common in children aged six to ten. Typical syndromes are headache, nausea, diarrhea and fever with red rash, and chronic anemia in HIV patients. Elevated serum anti-parvovirus B19 IgM and antibodies to the autoantigen IA-2 have been described with a homology in amino acid sequences between B19 and the extracellular domain of IA-2 [105]. The autoantigen IA-2 (islet cell antigen 512) is a member of the protein tyrosine phosphatase, secreted by the pancreatic endocrine cells and a regulator of insulin synthesis. A link between acute parvovirus B19 infection and T1D has been shown [132]. Parvovirus B19 can stimulate T-cell-mediated proliferative response. It activates autoimmunity by presentation of HLA class II antigen to CD4+ T-cells [106, 133]. Studies on the Kilham rat virus (KRV) belonging to the same genus have suggested molecular mimicry [134] and initiation of innate immunity in the pancreatic lymph nodes [135].* In vivo*,* in vitro,* and epidemiological evidence are required to define further the role of these viruses in T1D induction.

### 7.3. Rotavirus

Species* Rotavirus A* is the most common cause of childhood gastroenteritis and is suspected of triggering T1D. Association between rotavirus and T1D was shown by Honeyman et al. [136], who demonstrated specific seroconversion and increase in autoantibodies in T1D patients. In the experimental model, rotavirus infection caused inflammation of the insulin producing cells and induction of diabetes, which was attributed to *β*-cell autoimmunity [137]. The authors suggested a possible mechanism which involves increased exposure of *β*-cells to immune recognition and activation of autoreactive T-cells by proinflammatory cytokines. Rotavirus could induce or affect the islet autoimmunity by molecular mimicry because rotavirus contains peptide sequences, in VP7 (viral protein 7), highly similar to T-cell epitopes in the islet autoantigens glutamic acid decarboxylase-65 and tyrosine phosphatase IA-2 [107]. The published literature is insufficient to make any conclusive remarks with respect to the inductive role which rotaviruses have on T1D [138].

### 7.4. Rubella Virus

Infection by rubella virus during pregnancy has been related to increased risk of diabetes in the offspring suffering from congenital rubella syndrome. Congenital rubella was associated with induction of islet autoantibodies in 10% to 20% of congenital rubella cases with patients 5 to 25 years of age [108, 139]. Children who have rubella antibodies present before measles-mumps-rubella vaccination have been shown to have higher levels of islet cell autoantibodies than do seronegative children [140, 141]. Molecular mimicry has been suggested as the mechanism for the association of rubella virus with T1D induction, where the cross-reaction between glutamic acid decarboxylase and various rubella peptides by T-cells is involved [109]. Experimental* in vitro* studies and* in vivo* hamster models suggest direct *β*-cell infection and cytolysis. Rubella may fit the classical picture of viruses involved in the triggering of T1D. However, the reduced rubella infections after the introduction of the measles-mumps-rubella vaccination and the increase in T1D cases (an inverse relationship) in the world seem to be contradictory. The measles-mumps-rubella vaccination and autoantibody induction hypotheses are more likely to be related to T1D than are direct infection and cytolysis. More factors likely play a role in the onset, but more studies are required in this regard.

### 7.5. Mumps Virus

Mumps virus has demonstrated the ability to infect *β*-cells, leading to a decrease in insulin secretion in the human fetal cultured islet. The infection is associated with an increase of the HLA class I molecule expression, which could influence the autoimmune process in prediabetic individuals by increasing the activity of autoreactive cytotoxic T-cells [142]. Cavallo et al. [110] showed that the mumps virus infection was related to IL-1 and IL-6 release, so mumps could induce diabetes by decreasing tolerance toward *β*-cells and making them more sensitive to immune-mediated destruction.

A high number of children with mumps showed islet cell antigens for 2–15 months following the infection with this virus [143]. Another study associated the virus with an increase in the incidence of T1D [144]. A shared epitope, 7-amino-acid-long in the mumps virus nucleocapsid and in MHC class II molecule, has been suggested as the cause of immunological cross-reactivity between these molecules [111]. Regarding the rubella virus and measles-mumps-rubella vaccination, a similar suggestion has been made: mumps infections have decreased, while TD1 incidence has increased [144]. More investigations are required to shed light on the mumps virus studies.

### 7.6. Human Endogenous Retroviruses

Endogenous retroviruses (ERVs) represent the proviral phase of exogenous retroviruses that have integrated into the host cells. In humans, the most active endogenous retroviruses are members of Human endogenous retroviruses which are not included in the classification of the family* Retroviridae* [145], forming a part of the human genome. Retroviruses can integrate with the human genome so their genes are either inherited (derived from old viral infections of the germ cells) or acquired after birth. Environmental factors (diet, common viral infections, and/or the sex-hormone changes) may activate endogenous retrovirus genes which then work as a triggering factor [112]. Human endogenous retrovirus genes could be transcribed, expressed in protein, and responsible for the development of autoantibodies that might react against host proteins and these mechanisms could lead to T1D. Human endogenous retrovirus-K may influence the immune response through insertion close to or in neighboring genes involved in immune epigenetic regulation. Human endogenous retroviruses are known to induce proinflammatory cytokines production, as IL-1*β*, IL-6, or TNF-*α*, through cells, such as monocytes [146]. This virus may involve a combination of the epigenetic factor with other components in the triggering of T1D.

## 8. Special Viral Family:* Picornaviridae*

**Figure 2:**
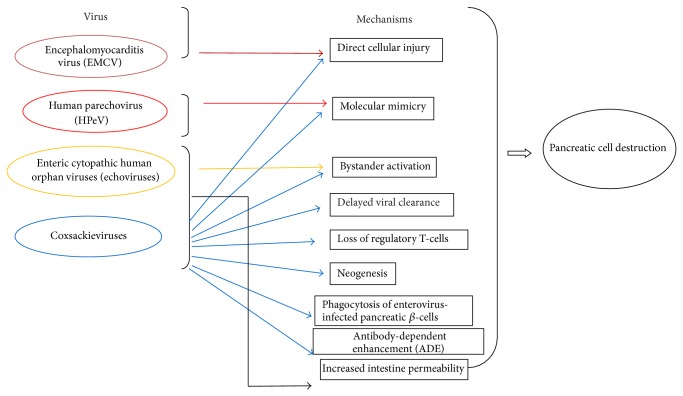
Putative mechanisms suggested for the induction by members of the family* Picornaviridae*. (Viruses linked to the induction of T1D in experimental animals (natural nonhuman hosts) or through clinical studies, having a potential to infect humans in rare conditions.) Factors influencing the mechanisms involved in the beta cell destruction by these viruses. (a) Genes related to infection putatively influencing the mechanisms: HLA-DR, melanoma differentiation-associated protein-5, and interferon induced helicase 1. (b) Innate immunity: interferons, tumor necrosis factor, and interleukins. (c) T- and B-lymphocytes, viral antibodies, islet cell antibodies, glutamic acid decarboxylase antibodies, antibody against insulin, and tyrosine phosphatase-related IA2-A antibodies.

Of the viral agents involved in presumptive triggering of T1D, the most studied ones belong to the family* Picornaviridae*. We will first describe the viruses that are linked to the induction of T1D in experimental animals (natural nonhuman hosts) but have the potential to infect humans in rare conditions. Then, we will describe the viruses that are shown to be associated with T1D in human clinical cases.  Figure 2 shows a schematic representation of the different viruses of this family and the suggested mechanisms for the destruction of pancreatic islets and function.

### 8.1. Encephalomyocarditis Virus

Encephalomyocarditis virus belongs to the genus* Cardiovirus* of the* Picornaviridae* family. The common hosts of these viruses are rodents and pigs although the virus can infect any mammal. Recently these viruses were shown to circulate naturally in humans in South America [147]. The clinical symptoms in humans usually go unnoticed, but in serious cases symptoms include high fever, nausea, headache, rigidity, delirium, vomiting, photophobia, and pleocytosis.

These viruses are able to induce the rapid onset of diabetes in mice. Two main variants of encephalomyocarditis virus have been determined: the nondiabetogenic variant encephalomyocarditis virus-B and the diabetogenic variant encephalomyocarditis virus-D, both with tropism for pancreatic *β*-cells. There are differences in 14 nucleotides and in the 776th amino acid, alanine (Ala-776), of the encephalomyocarditis virus polyprotein, located at major capsid protein VP1. These changes are seen only among all diabetogenic variants. In contrast, threonine in this position (Thr-776) is observed particularly in all nondiabetogenic viruses. The tyrosine kinase-2 gene expression prevented beta cell destruction by encephalomyocarditis virus-D in knock-out mice [148]. The prevention of macrophage-related cell destruction was shown to be induced by knocking out the tyrosine kinase pathway.

### 8.2. *Human parechoviruses*

Both species* Human parechovirus* (some of which are former echovirus serotypes) and Ljungan virus which belong to genus* Parechovirus* have been implicated in the development of T1D [113]. These viruses can naturally infect bank voles but are also known to cause infections in humans. T1D was described in the animals after one month of observation in the laboratory. The symptoms were persistent hyperglycemia with weight loss, ketosis, and hyperlipidemia as well as specific *β*-cell destruction associated with signs of autoimmunity (increased levels of autoantibodies to glutamic acid decarboxylase-65, autoantigen IA-2 (islet cell antigen 512), and insulin). The disease was correlated with Ljungan virus antibodies. Antibodies to Ljungan virus have been shown in children with onset of T1D and a possible zoonotic infection has been proposed [149]. The virus could be involved in T1D but more epidemiological and experimental studies are required to elucidate the mechanisms involved.

### 8.3. Enteric Cytopathic Human Orphan Viruses

These viruses, commonly referred to as echoviruses, belong to genus* Enterovirus*,* Enterovirus B* species. An enteric cytopathic human orphan virus strain isolated from a 6-week-old baby suffering from acute T1D [114] was shown to be more destructive in human islets* in vitro* than the echovirus 9 and 30 other prototype strains [150]. Cabrera-Rode et al. [151] detected the presence of insulin antibodies, glutamic acid decarboxylase antibodies, and autoantigen IA-2 (islet cell antigen 512) in the serum indicating that the islet cell autoimmunity was associated with infection in the year 2003 aseptic-meningitis Cuban epidemic caused by echovirus 16. During an echovirus 30 epidemic in Cuba, Cabrera-Rode et al. [152] reported the case of an adolescent who developed pancreatic autoantibodies (ICA and IA2A) and T1D after infection. Diaz-Horta et al. [153] provide a list of autoantibodies produced following various echovirus infection. In short, islet cell autoantibodies were related to echoviruses 3, 6, 9, 16, and 30. Glutamic acid decarboxylase-65 was related to echoviruses 6 and 16; autoantigen IA-2 (islet cell antigen 512) was linked to echoviruses 3 and 16; and insulin antibodies were related to echoviruses 9 and 16 [114, 116, 151, 152, 154–156]. The published literature shows strong association between echoviruses and T1D onset.

### 8.4. Coxsackieviruses

Coxsackieviruses belong to the genus* Enterovirus*, species* Enterovirus A* and* Enterovirus B*. The coxsackievirus type B consists of 6 serotypes that have been investigated* in vivo* and* in vitro* to establish association with chronic diseases since an early report of a link among coxsackievirus infections, myocarditis [157], and T1D [158]. The most often studied enteroviruses are coxsackieviruses B, as they have been diagnosed frequently from clinical samples of patients with T1D (or high risk thereof) compared to a healthy population. Several seroepidemiological studies have demonstrated that recent-onset diabetic patients had increased levels of coxsackievirus-specific antibodies or coxsackievirus-RNA compared to control populations [116, 159–161]. Sarmiento et al. [162] also found this correlation in Cuba, where the incidence of T1D is low [163] but coxsackieviral infections incidence is high. Coxsackievirus B4 (CVB4) has also been directly isolated from the pancreas autopsy of a recent-onset T1D patient [164].

### 8.5. Enteroviral Mechanisms for T1D Predisposition or Protection

Coxsackieviruses are under intense scrutiny after the first isolation reported in 1979 [164]. They have been demonstrated to accelerate diabetes in genetically susceptible mouse models [117, 118, 165–167]. Different* in vivo* and* in vitro* coxsackievirus models are described in Table 5. Prospective and cross-sectional studies of patients published in the year 2014, from Finland, England, France, Greece, and Sweden, [168, 169] showed two different mechanisms within the different serotypes of coxsackieviruses. One was destructive and the other protective, consistent with results from the nonobese diabetic (NOD) mouse model of coxsackievirus B-associated T1D. Coxsackievirus B1 (CVB1) was shown to be related to autoimmune destruction of pancreatic *β*-cells. They have shown that virus neutralizing antibodies appeared only a few months before the autoantibodies and were related to the presence of maternal antibody modification, whereas the other prevalent viruses CVB3 and CVB6 showed a protective effect.

Experimental* in vivo* and* in vitro* models show protection or destruction of the pancreatic islets and the endocrine function in response to virus infection. In these models the influencing factors* in vivo* are the host's age at the time of infection, host and virus genetics, and dose of infection. In our experimental coxsakievirus serotype B4 strain E2 (CVB4-E2) infection model using outbred gravid mice [170], we showed gross infiltration of the endocrine pancreas and glycemia in the virus-challenged pups weaned from dams infected at the 1st and 3rd weeks of gestation. Another group showed that maternal infection conferred protection to the offspring from diabetes in a transgenic* Socs1-tg* mouse model, with nonobese diabetic (NOD) genetic background [171]. Evidence indicated that the time of infection during the pregnancy and age of the pups at time of challenge are important factors. To determine the conditions for protective or destructive outcomes further studies are required to elucidate the mechanisms.

### 8.6. Coxsackievirus* In Vitro* Studies

Coxsackievirus serotypes have been shown to replicate and destroy human *β*-cells [150, 172, 173] and mouse islet *β*-cells [174]* in vitro.* Prolonged persistence of the virus in human pancreatic islet cells has also been shown* in vitro* [126, 175]. Rodent *β*-cells are resistant to metabolic disturbances caused by the prototype strain CVB4 [115, 176, 177]. Porcine endocrine cells have also been used as models for virus infection and for studying the pathogenesis of T1D [176] (due to their cost). Porcine islet cells are susceptible to virus-induced impairment but are relatively resistant to oxidative, toxins, and cytokine-induced damage [178]. Direct cytolysis of the islet cells which involves infection of the *β*-cells, replication of the virus resulting in lysis of the cells, depends on the genetics of the virus.

### 8.7. Coxsackieviruses in Human Samples of T1D

Coxsackie B viruses were linked to T1D when a coxsackievirus B4 (CVB4) strain was isolated from the pancreas during the autopsy of a 10-year-old child who had died from diabetic ketoacidosis. In the pancreatic tissue necrosis of *β*-cells with infiltration of lymphocytes was seen. Inoculation of mice with the viral isolate resulted in hyperglycemia on day 5 after infection and inflammation of the islets of Langerhans and necrosis to day 14 after infection only in SJL mice after two passages of the virus* in vivo* [164].

There is also serological evidence in pregnant women with coxsackievirus (incidence of anti-enteroviral antibodies in the sera of pregnant women) and subsequent development of T1D in the children [179–181]. There is however one report that does not support this finding [182]. These differences maybe related to the time of infection during gestation.

The enterovirus (EV) genome and antibodies against CVB1-B6 can be found in sera of prediabetic children several years before the onset of diabetic symptoms, which have been associated with induction of autoimmunity. Studies of sera from newly diagnosed diabetics have revealed increased levels of anti-EV neutralizing antibodies (as compared with controls) [183–186] and elevated T-cell responses to coxsackievirus antigen [187].

Numerous researchers have detected antiviral antibodies and viral-RNA in blood/serum/peripheral blood mononuclear cells by PCR, sequencing methods, or in tissues of postmortem pancreatic specimens with* in situ* hybridization and identification of viral proteins by immunohistochemical staining from T1D patients.

Enteroviral-RNA was associated with an increase in antibodies against islet cells (ICA) and glutamic acid decarboxylase (GAD) [116, 162] as well as antibodies against insulin (IAA) or the tyrosine phosphatase-related IA-2 protein (IA-2) [188]. The enteroviral infections are prevalent in children who became positive for *β*-cell autoantibodies compared to healthy controls [116, 160, 162, 188–190].

Enteroviral-RNA has also been shown in the whole blood of patients at the onset or during the course of T1D but was not shown in healthy subjects and patients with T2D. Sequencing of circulating enteroviral-RNAs in T1D patients has confirmed systemic viral coxsackievirus infection [159]. In another study, the presence of IFN-*α* mRNA was detected by reverse transcriptase polymerase chain reaction (RT-PCR) in whole blood and sera of the T1D patients but in none of controls. Enteroviral-RNA was detected in patients' blood samples with IFN-*α* but not in patients without any IFN-*α*. Circulating enterovirus-RNA was sequenced by Chehadeh et al. [97]. Enteroviral-RNA in blood spots (taken on days 2–4 after birth for screening analysis of inherited metabolic diseases) showed increased prevalence of enteroviral-RNA in children preceding the T1D [191]. Coxsackievirus B4-specific RNA was found in the sera of diabetic children [186, 192, 193] and a significant proportion of diabetic children with a positive RT-PCR were even less than one year old [194, 195]. In the peripheral blood mononuclear cells enteroviral-RNA was detected by RT-PCR [196] and enteroviral capsid antigens were detected by immunofluorescence [197]. Prolonged enterovirus infections could be found in patients who were positive for the detection of viral-RNA in peripheral blood mononuclear cells and/or plasma together with the absence of viral-RNA in stool and throat swabs [198].

Enteroviral-RNA is commonly found in stool samples of T1D children [190, 199, 200], but a direct connection to the onset of T1D and acute coxsackievirus infection and the presence of enterovirus in the stool remains uncertain.

Different virological methods have been used to determine whether enteroviruses can be found in small intestinal mucosa of T1D patients undergoing intestinal biopsies. Viral-RNA was found by RT-PCR in a frozen sample. In these samples protein VP1 was localized in the epithelium by immunohistochemistry and enteroviral-RNA was detected by* in situ* hybridization in the cells of lamina propria [122].

Enterovirus-positive cells have been detected in numerous pancreatic islets and some duct cells with nondestructive insulitis and natural killer cell infiltration but were not seen in the exocrine pancreas [201]. Using electron microscopy Dotta et al. [202] observed viral inclusions and signs of pyknosis and loss of *β*-cell function. In different studies using immunohistochemistry for VP1 protein was demonstrated in endocrine cells of the pancreatic islets during autopsies of diabetic children [119, 202–204]. Although the enteroviruses have been found in the pancreatic islets of patients with recent-onset of diabetes [202, 204, 205], *β*-cell destruction in patients with fatal diabetes was not a direct outcome of the virus-mediated cytopathic effect. Viruses induce *β*-cells destruction through an autoimmunity mechanism.

## 9. Viruses: Mechanism of Protection Hypothesis

Type 1 diabetes cases are increasing worldwide, especially in highly industrialized countries and urban regions. Such statistics have given rise to the “hygiene hypothesis” which was originally proposed to explain increases in asthma and allergic diseases [206]. Since the year 1989, the hygiene hypothesis has also been suggested as an explanation of increased rates of other autoimmune diseases including T1D [207]. A lack of exposure to a wide range of infectious agents in industrialized or developed countries is presumed to increase the rates of some diseases especially those which are transmitted by the fecal-oral route. While the incidence of various infectious diseases has decreased over the last few decades, the occurrence of autoimmune disorders has increased rapidly [207]. Several authors have discussed the protective effect of the enteroviruses on T1D [208–211]. Serological and epidemiological studies and* in vivo*/*in vitro* models indicate that the effect of virus on the pancreatic islet cells affliction/protection depends on several factors.

## 10. Viruses: Mechanism of ***β***-Cells Destruction

Enterovirus infections often go unnoticed, and humans may be infected several times during their lifetime with different enteroviruses. Recent studies show that viral-RNA may persist over a long period of time. These viruses have a wide range of tropism, they induce interferons, their teratogenic character is vague as compared with other viruses, and they may cause multiple infections and immune imbalances in the defence system. Epigenetics and other factors such as age of host at the time of infection, multiple infections, maternal infections, circulating viruses genetics, and a combination of other factors have resulted in different/controversial opinions. The actual mechanism could be a destructive pathway which is a combination of different factors. One has to consider that the methodologies applied here are for detection of RNAs or for analyzing viral genome persistence.* In situ* hybridization and VP1 detection need standardized methodology. Different mechanisms related to enterovirus are described below.

### 10.1. Direct Cellular Injury

Human and prototype laboratory coxsackieviruses A and B strains can infect mouse islet cells* in vitro* [174]. In addition, human islet *β*-cell infection* in vitro* and its destruction by enteroviruses and clinical isolates have been recorded [85, 150, 212–215]. Pathogens, mainly viruses that infect pancreatic islet cells, may induce T1D through direct cytopathic effects and cause destruction of the insulin-secreting *β*-cells by cytolysis; enteroviruses are known to be highly cytolytic [172, 216]. Direct infection causes disruption of cells and release of autoantigens which activate the innate and adaptive immune systems. The ensuing inflammation and development of autoimmune reactions further contribute to *β*-cell destruction and T1D.

### 10.2. Delayed Viral Clearance

Delayed viral clearance is based on evidence that individuals with high genetic risk for T1D have impaired defence mechanisms against virus clearance [217]. Virus persistence in the pancreatic *β*-cells can result in the induction of autoimmunity. In enterovirus-infected individuals the viremia is short, yet enteroviral-RNA has been found in the blood of patients with newly diagnosed T1D which may be a result of prolonged enteroviral persistence in blood cells [198]. Enteroviruses detected at the onset of T1D were not found in plasma but were present in peripheral blood mononuclear cells. The viruses might use monocytes and/or lymphocytes as viral reservoirs and vehicles for viral dissemination [218]. T-cells from diabetic patients show reduced activation and cytokine production when challenged* in vitro* with coxsackievirus B4 [217]. Patients with coxsackievirus infection with insufficient immune response to provide total protection, maybe at a high risk of delayed virus clearance, viral persistence, and increased pancreatic islet damage, are high risk patients. Many T1D patients show detectable levels of viral-RNA suggesting that a delayed clearance may be more relevant than viral persistence to the progression of T1D [116].

### 10.3. Molecular Mimicry

Molecular mimicry is based on the observation that some microbial/viral proteins and host proteins have sequence or structural homology and therefore go unrecognized as self-proteins. A normal immune response against the viral antigen becomes cross-reactive against the homologous sequence of the *β*-cells host protein [219]. Glutamic acid decarboxylase-65 (GAD65) expressed in pancreatic *β*-cells is the important islet cell autoantigen which can be recognized as nonself in T1D [114]. There is a structural similarity between P2-C protein sequence of CVB and an epitope derived from the humans and NOD mice, the GAD65 [220]. Therefore, both autoreactive and antiviral T-cells activated upon CVB infection might act as strong enhancers of the autoimmune process. Enteroviral infections can activate the antienteroviral T-lymphocytes which, via cross-reactivity, contribute to the damage of *β*-cells persistently infected by another enterovirus [154]. Some* in vivo* and* in vitro* studies [118, 221, 222] do not support the theory of molecular mimicry due to lack of cross-reacting glutamic acid decarboxylase epitopes and viral antigens.

### 10.4. Bystander Activation

Infection of cells neighboring the *β*-cells (e.g., ductal cells) may stimulate local inflammation. As a consequence CD8+ T-cells and inflammatory cells (macrophages) release cytokines such as tumor necrosis factor alpha, lymphotoxin, and nitric oxide, which can lead to bystander killing of *β*-cells [223]. The inflammation provokes cell damage and release of segregated antigens. Through bystander activation the T-lymphocytes directed against these self-antigens are responsible for pancreatic islet destruction and T1D development [224–226]. Diabetes develops when the damage gets out of control. In contrast to molecular mimicry, initial tissue injury via bystander activation is not antigen specific as the T-cells have not been shown to respond to the coxsackievirus. The injury is due to inflammation via the bystander activation of cells [118]. Viral infection can also lead to the activation of antigen-presenting cells, namely, dendritic cells (DCs). Activated APCs then increase T-helper density at the site of infection/inflammation [227]. Horwitz et al. [228] and Serreze et al. [117] suggested that the number of autoreactive T-cells is important for T1D induction after coxsackievirus B4 infection for initiation of bystander activation.

### 10.5. Antibody-Dependent Enhancement (ADE) of Enterovirus Infection

Antibodies are essential in limiting, clearing, and influencing the severity of enteroviral infections. This mechanism is mediated by anti-coxsackievirus antibodies lacking neutralizing activity. These antibodies bind to the cell surface membrane of the monocytes/macrophages via the coxsackie-adenovirus receptor (CAR) and increase the replication of CVB4 in the cells. The infected monocytes/macrophages help virus dissemination in the host, as well as viral replication target sites enhancing pathological responses, known as antibody-dependent enhancement. This pathological severity was shown to be enhanced (*in vitro*) when plasma or IgG from patients with T1D was used as compared to those of healthy individuals [229]. Coxsackievirus B4 induces interferon-*α* in peripheral blood mononuclear cells* in vitro* [230, 231], and chronic IFN-*α* synthesis or its abnormal activation can be associated with disorders leading to autoimmune diseases [97]. These antibodies target the EV-protein VP4 and it has been shown that the prevalence and anti-VP4 antibody titres are in higher concentrations in patients with T1D than those in control subjects [232, 233].

### 10.6. Phagocytosis of Enterovirus-Infected Pancreatic *β*-Cells

Another suggested mechanism in which virus infected islets may affect the local immune balance has been shown* in vitro* in human islets [120] where coxsackievirus-infected islets were efficiently phagocytosed by human monocyte-derived dendritic cells. Phagocytosis induces an antiviral state that protects dendritic cells from subsequent coxsackievirus infection. This antiviral state of protected dendritic cells depends on the presence of intracellular viral-RNA in the coxsackievirus-infected cells and type 1 interferons produced by the dendritic cells. It has been suggested that these virus-induced effects may alter dendritic cells, therefore influencing the development of regulatory T-cells and/or effector T-cell populations.

### 10.7. Loss of Regulatory T-Cells

The failure of immunological tolerance towards *β*-cell antigens after the T-cell and B-cell maturation is due to abnormalities of the T-regulatory lymphocytes (Tregs) in the periphery. This occurs outside the primary lymphoid tissues along/only with the central tolerance and involves the thymus [234]. Coxsackievirus B4 replicates and persists in human thymic epithelial cells* in vitro* [121] as well as mouse* in vitro* and* in vivo* models [235, 236], which disturbs maturation/differentiation of T-lymphocytes. Enteroviral infection of thymus has been suggested to result in defective T-lymphocyte subpopulations which have been found in diabetic and prediabetic patients [237].

### 10.8. Increased Intestinal Permeability

Enteroviruses have been detected in small intestinal biopsies of T1D patients more frequently than in healthy controls, suggesting persistence of enterovirus infections and replication in the gut for prolonged periods [122]. Increased intestinal permeability as an outcome of prolonged infections, which could be associated with an increased susceptibility to T1D, has also been suggested [122, 238]. Enteroviral infections lead to changes in gut permeability and increased viral access to the pancreas allowing other environmental factors to modify T1D susceptibility.

### 10.9. Neogenesis

Pancreatic ductal cells can differentiate into functioning adult *β*-cell mass [239]. Normally a stable rate of recirculation of “apoptotic *β*-cell replacement” takes place during the differentiation of progenitor cells [240]. It has been suggested that coxsackievirus infections afflict *β*-cell neogenesis causing depletion of *β*-cell mass which would have a role in diabetes development [123].

## 11. Conclusion

The incidence of T1D has increased rapidly in recent years. Whether the increase is an outcome of synergistic influence(s) of different factors is unclear. The onset of T1D is a coordination of multiple factors. Several risk/protective elements are, however, associated with the incidence of T1D: family history, host genetics, immunological status, sex, age, obesity, ethnicity, and social group characteristics, as well as behavioral, lifestyle, psychological, and clinical factors. Furthermore the T1D process may start in the early neonatal stage or even* in utero*, and the environmental factors encountered in early childhood might also induce or accelerate the disease [155]. Among such influential conditions are exposure to intrauterine infections, nutrition, and consumption of toxic material. The time of exposure during gravidity and delivery conditions may also be relevant [241].

The genetic predisposition depends mainly on the MHC and non-MHC genes, which are proven major factors in favoring T1D induction. These genes direct the immune responses which are important in autoimmune diseases.

Suggested mechanisms for triggering T1D consider direct or indirect interaction of viruses and immune system in genetically predisposed individuals. Viruses may cause variation in certain genes of the MHC loci and may upregulate or dysregulate inflammatory or proinflammatory cytokines leading to destructive or protective effect on pancreatic islet cells. Numerous investigations on the pathogenesis of T1D and the involvement of viruses have been carried out. However, the onset of T1D and the triggering factor/s and mechanisms involved remain unsolved. Together, various types of studies (observational and experimental) indicate that the mechanism of T1D indication via genetic-viral interactions is complex.

## Figures and Tables

**Table 1:** Current and former classifications of diabetes mellitus.

Former classification (based on treatment)	Current classification	Cause^*^
Insulin-dependent diabetes mellitus (IDDM)	Type 1 diabetes (T1D)	*β*-cell destruction that leads to total insulin deficiency

Non-insulin-dependent diabetes mellitus (NIDDM)	Type 2 diabetes (T2D)	A progressive defect in insulin secretion in combination with insulin resistance

Heterogenic	Other types of diabetes: maturity-onset diabetes of the young (MODY), neonatal diabetes mellitus (NDM), genetic syndrome associated with diabetes	Genetic defects in function of the *β*-cells. Other factors include pathophysiology of the pancreas (cystic fibrosis or pancreatitis) and drug- or chemical-induced diabetes in patients. Syndromes: Down syndrome, Huntington's chorea, Prader-Willi syndrome, diabetes insipidus, Rabson-Mendenhall syndrome, and immune-mediated disorders such as systemic lupus erythematosus

Heterogenic	Gestational diabetes (GD) diagnosed during pregnancy	Related to hormonal changes, low insulin levels, nutritional and genetic factors

^*^Causes or current definitions as per [7–9].

**Table 2:** The major histocompatibility complex and the human leukocyte antigens^*^.

	HLA loci			Combinations most associated with T1D	Cell type	Functions
MHC I	A			A24	Mainly cytotoxic CD8+ T-cells, also other types of nucleated cells	Peptide binding protein for antigen presentation
	B			B8, B18, B39		
	C					Processing of antigens

MHC II	DP	DP-alpha	DP*α*1		Helper CD4+ antigen-presenting cells (dendritic cells, macrophages, some endothelial cells, thymic epithelial cells, and B-cells)	Peptide binding protein
		DP-beta	DP*β*1	DP*β*1		
	DQ	DQ-alpha	DQ*α*1			
		DQ-beta	DQ*β*1			
	DR	DR-alpha				Helper activity for specific MHC II proteins
		DR-beta	DR*β*1	DR*β*1, DQ*β*1, DR-DQ combinations, DR*β*1-DQ*α*1-DQ*β*1 subset combinations		
			DR*β*2			
			DR*β*3			
			DR*β*4			

MHC III	Are flanked by the MHC I and MHC II coding regions			Novel area for research	Various	Components of the complement systems C2, C4a, and C4b Cytokines Heat shock proteins

MHC IV	Exist at the telomeric end of MHC III genes			Novel area for research	Dendritic cells, natural killer cells	Morphogenesis of dendritic cells, natural killer cells

^*^References [9, 29–36, 41–50].

**Table 3:** Nonmajor histocompatibility complex genes and gene products associated with type 1 diabetes by genome wide association studies.

Chromosome^*^ number	Gene^*^	Function of the gene product
Chromosome 1	Protein tyrosine phosphatase nonreceptor 22 gene (PTPN22)	Plays a role in T-cell receptor signaling
Chromosome 1	Interleukin-10 (IL-10)	Downregulates expression of MHC II antigens and Th1 cytokines and is involved in cell mediated and cytotoxic inflammatory response
Chromosome 2	Including cytotoxic T-lymphocyte antigen 4 (CTLA4)	Expressed by CD4+ and CD8+ T-cells and downregulates T-cell proliferation and cytokine production
Chromosome 2	Interferon induced with helicase C domain 1 (IFIH1/MDA-5)	Triggers the secretion of interferons in response to viral infections
Chromosome 5	Interleukin-4 (IL-4)	Induces differentiation of naive T-cells to T-helper cells but suppresses interferon-*γ* and IL-2 producing Th1 cells
Chromosome 5	Interleukin-12 beta also known as interleukin-12p40 (IL-12B)	Produced by antigen-presenting cells and drives the differentiation of CD4+ T-cells into Th1 cells
Chromosome 6	Small ubiquitin-like modifier 4 (SUM04)	Polymorphism in this gene leads to activation of nuclear factor kappa B
Chromosome 6	Tumor necrosis factor alpha (TNF-*α*)	Stimulates inflammatory reactions and phagocytosis
Chromosome 7	Paired box gene 4 (PAX4)	Plays a role in tissue development and is found on pancreatic islet cells
Chromosome 10	IL-2 receptor-alpha (IL-2RA)	Its expression on T-cells is necessary for suppressing T-cell response
Chromosome 11	Insulin (INS)	Controls glucose levels in the blood
Chromosome 12	2′-5′-Oligoadenylate synthetase 1 (OAS1)	Enzyme involved in the innate immune response induced by interferons against viral infections
Chromosome 12	Interferon gamma (IFN-*γ*)	Cytokine involved in the inflammatory responses, produced by different natural killer cells, CD4+, and CD8+ T-cells
Chromosome 13	Interferon-regulating factor 7- (IRF7-) driven inflammatory network (IDIN)	Present in monocytes and regulated by viral responses
Chromosome 16	C-type lectin domain family 16 (CLEC16A)	Expressed in most of the immune cells and plays a role in the antigen uptake
Chromosome 18	Tyrosine protein phosphate nonreceptor 2 gene (PTPN2)	Regulates proinflammatory cytokines

^*^References [25–27, 29, 63–81].

**Table 4:** An overview of the viruses associated with T1D and their classification.

Viruses	Species	Genus	Family	Genome
Human cytomegalovirus	*Human cytomegalovirus *	*Cytomegalovirus *	*Herpesviridae *	dsDNA
Parvovirus B19	*Primate erythroparvovirus 1 *	*Erythroparvovirus *	*Parvoviridae *	ssDNA
Kilham rat virus	*Rodent protoparvovirus 1 *	*Protoparvovirus *	*Parvoviridae *	ssDNA
Rotavirus	*Rotavirus A *	*Rotavirus *	*Reoviridae *	dsRNA
Rubella virus	*Rubella virus *	*Rubivirus *	*Togaviridae *	Positive ssRNA
Mumps virus	*Mumps virus *	*Rubulavirus *	*Paramyxoviridae *	Negative ssRNA
Human endogenous retrovirus			*Retroviridae *	ssRNA
Encephalomyocarditis virus-K	*Encephalomyocarditis virus *	*Cardiovirus *	*Picornaviridae *	Positive ssRNA
Parechovirus	*Human parechovirus *	*Parechovirus *	*Picornaviridae *	Positive ssRNA
Echovirus	*Enterovirus B *	*Enterovirus *	*Picornaviridae *	Positive ssRNA
Coxsackievirus	*Enterovirus B *	*Enterovirus *	*Picornaviridae *	Positive ssRNA

**Table 5:** Viruses linked with type 1 diabetes in humans.

Virus	Family	Mechanism of the T1D induction	Model system
Human cytomegalovirus	*Herpesviridae *	Persistent infection [102]	Lymphocytes and autoantibodies (clinical study)
		Molecular mimicry [103]	GAD65-specific T-cells cross-react with a peptide of the HCMV (*in vitro*)
		Bystander activation [104]	Activation of CD4+ and CD8+ T-cells (clinical study)

Parvovirus	*Parvoviridae *	Molecular mimicry [105]	Elevated serum anti-parvovirus B19 IgM and autoantibodies (clinical study)
		Induction of autoimmunity [106]	Prolonged autoimmune alterations (clinical study)

Rotavirus	*Reoviridae *	Molecular mimicry [107]	Correlation in the proliferative responses of T-cells to the similar peptides in rotavirus and islet autoantigens (*in vitro*)

Rubella virus	*Togaviridae *	Congenital infection [108]	Children with congenital rubella-autoantibodies (clinical study)
		Molecular mimicry [109]	T-cell response to viral and beta cell peptides (*in vitro*)

Mumps virus	*Paramyxoviridae *	Loss of tolerance toward *β*-cells [110]	Human insulinoma cell line infected with mumps (*in vitro*)
		Molecular mimicry [111]	Antibodies in serum of vaccinated and nonvaccinated children (clinical study)

Human endogenous retrovirus	*Retroviridae *	Influence of the immune response [112]	Presence of antigen in T-cell subsets of patients (clinical study)

Human parechovirus	*Picornaviridae *	Induction of autoimmunity [113]	Stool samples and autoantibodies (clinical study)

Echovirus	*Picornaviridae *	Molecular mimicry [114]	Echovirus 9 isolated from baby was destructive for human islets* (in vitro) *

Coxsackievirus	*Picornaviridae *	Direct cellular injury [115]	CVB4 and SJL/J mice (*in vivo*)
		Delayed viral clearance [116]	Serum of prediabetic children (clinical study)
		Molecular mimicry [117]	Autoantibodies in human blood samples (clinical study)
		Bystander activation [118]	CVB4 and NOD and BDC2.5 mice (*in vivo*)
		Antibody-dependent enhancement [119]	CVB4 and human serum-PBMC and monocyte (*in vitro*)
		Phagocytosis of infected *β*-cells [120]	CVB3-infected human and porcine pancreatic islets (*in vitro*)
		Loss of regulatory T-cells [121]	CVB4-E2 infection of human thymic epithelial cells (*in vitro*)
		Increased intestine permeability [122]	Virus presence in the small intestine biopsy samples
		Disruption in *β*-cells neogenesis [123]	CVB4-E2 or CVB4-JVB and SJL/J mice
